# Improving Focal Photostimulation of Cortical Neurons with Pre-derived Wavefront Correction

**DOI:** 10.3389/fncel.2017.00105

**Published:** 2017-05-01

**Authors:** Julian M. C. Choy, Sharmila S. Sané, Woei M. Lee, Christian Stricker, Hans A. Bachor, Vincent R. Daria

**Affiliations:** ^1^John Curtin School of Medical Research, Australian National UniversityCanberra, ACT, Australia; ^2^Research School of Engineering, Australian National UniversityCanberra, ACT, Australia; ^3^Medical School, Australian National UniversityCanberra, ACT, Australia; ^4^Research School of Physics and Engineering, Australian National UniversityCanberra, ACT, Australia

**Keywords:** adaptive optics, two photon microscopy, spatial light modulator, two-photon photolysis

## Abstract

Recent progress in neuroscience to image and investigate brain function has been made possible by impressive developments in optogenetic and opto-molecular tools. Such research requires advances in optical techniques for the delivery of light through brain tissue with high spatial resolution. The tissue causes distortions to the wavefront of the incoming light which broadens the focus and consequently reduces the intensity and degrades the resolution. Such effects are detrimental in techniques requiring focal stimulation. Adaptive wavefront correction has been demonstrated to compensate for these distortions. However, iterative derivation of the corrective wavefront introduces time constraints that limit its applicability to probe living cells. Here, we demonstrate that we can pre-determine and generalize a small set of Zernike modes to correct for aberrations of the light propagating through specific brain regions. *A priori* identification of a corrective wavefront is a direct and fast technique that improves the quality of the focus without the need for iterative adaptive wavefront correction. We verify our technique by measuring the efficiency of two-photon photolysis of caged neurotransmitters along the dendrites of a whole-cell patched neuron. Our results show that encoding the selected Zernike modes on the excitation light can improve light propagation through brain slices of rats as observed by the neuron's evoked excitatory post-synaptic potential in response to localized focal uncaging at the spines of the neuron's dendrites.

## 1. Introduction

Laser microscopy is an important tool to understand the fundamental processes of neurons in brain tissue. However, it remains a demanding task to probe neurons at deeper regions due to the degradation of the laser's coherent properties as it propagates through the tissue. While two-photon (2P) microscopy uses a less absorptive near-infrared (NIR) laser, refractive index inhomogeneity and scattering are still important factors that distort the point spread function (PSF) of the incident coherent light. Aside from affecting the resolution, the broadening of the PSF drastically reduces the probability of non-linear 2P absorption and consequently decreases the emitted fluorescence signal. While the fluorescence signal can be improved by increasing the average power of the excitation laser, such move can degrade living biological samples due to photo-toxicity and heating (Gautam et al., 2015), which can affect the physiological activity and are hence disruptive when probing fundamental cellular processes. Fluorescence imaging and photostimulation (e.g., photolysis of chemically caged compounds and optogenetics) will all suffer the same predicament. Hence, the key to achieve minimally invasive imaging and probing of cellular processes is to build-up the capacity to rectify distortions of the incident light field, which optimizes the ability to achieve a near diffraction-limited focus at deep regions of the brain tissue.

Although healthy brain tissues are made up of a heterogeneous distribution of neurons, dendrites and axons, these tissues are generally made-up of specific organization of cells. For example in the neocortex, a diverse spread of neurons organize into several layers forming the neuronal circuitry for information processing in the brain (Bullmore and Sporns, 2012). Such organization of the neurons and its neurites presents a predicable framework for mapping brain regions and visually targeting specific cells (e.g., Layer 2/3 and Layer 5 pyramidal neurons).

Earlier reports of optical imaging through optically thick brain tissues has paved the way for our investigations of neurons in their natural environment. When imaging optically thick brain tissues, light encounters multiple scattering and deviates from its original path. The scattered optical paths could be treated as almost deterministic and, when measured accordingly, it can be possible to build a transmission matrix (Yaqoob et al., 2008) or phase map (Schwertner et al., 2004) to correct for the light distortion in that particular instance. As such, recent techniques into the measurement and reversal of optical distortion in tissues (Booth, 2007) based on photo conjugation (Yaqoob et al., 2008), turbid layer conjugation (Park et al., 2015), sensorless imaging (Ji et al., 2010; Wang et al., 2014), artificial guide star (Tao et al., 2011a) and wavefront sensors (Tao et al., 2011b) have shown promising results. These techniques correct for the light distortion by treating sections of the tissue as distinct transmission matrices and hence require pre-measurement of a corrective wavefront for the incoming light prior to obtaining a wavefront corrected high-resolution image. In general, such active measurement works well for biological samples that do not deteriorate with time. However, as for many experiments involving living cells, when the window of time for the right experimental conditions is limited, an *a priori* identification of an appropriate wavefront correction is necessary.

In this work, we show that we can correct for light distortions by a pre-derived wavefront correction that is specific to particular regions in optically thick brain tissue. This allows us to pre-correct for light distortion without any wavefront sensing (Schwertner et al., 2004) and by using predictable Zernike modes measured *a priori*. While light experiences multiple scattering in tissues, differential interference contrast (DIC) imaging of these tissues relay that they still converge to form perceptible images giving us information of the cellular network. As cells within tissue can be distinguished by changes in refractive index, the cellular network can form visible and highly organized structures of varying refractive indices that manifest as optical aberrations for the incoming light. We can therefore correct for these aberrations using Zernike modes.

For the experimental application, we require rapid optimization of the laser focus through freshly prepared parasagittal brain slices from a rat for *in vitro* opto-electrophysiological experiments. Before performing experiments with living cells, we first used fixed tissue samples to identify Zernike modes that persistently optimize the focus at different locations within a selected brain region. It was apparent from the iterative procedure that a small subset of the modes can be used to optimize the focus. We then used these modes to improve the efficiency of 2P photolysis along dendrites of neurons embedded within brain slices. Two-photon photolysis releases chemically caged neurotransmitters (glutamate) near dendritic spines, thus emulating synaptic inputs to the neuron (Callaway and Katz, 1993; Denk, 1994). We show that there is an optimum uncaging response on a select set of Zernike modes encoded on the excitation light. Using just these few pre-determined Zernike modes allows the wavefront correction to be made with a significantly reduced optimization procedure, which is advantageous in time-critical experiments where the lengthy search for an ideal wavefront correction is not applicable.

## 2. Methods

### 2.1. *A priori* identification of zernike modes

**Figure 1:**
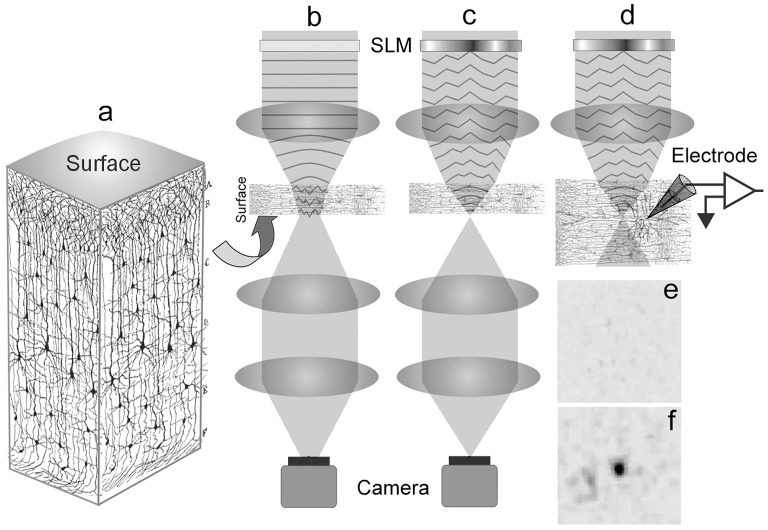
**Schematic of the experiment. (a)** A 3D visualization of a cortical brain slice (adapted and modified from Ramón y Cajal, 1909; Courtesy of the Cajal Institute-CSIC, Madrid, Spain ©CSIC), showing the organization of the neuropile. **(b)** Uncorrected light is scattered as it enters the brain tissue thus broadening the focus. **(c)** The spatial light modulator is encoded with a corrective wavefront to compensate the aberrations introduced by the tissue, resulting in a sharp focus. In **(b,c)** the focus at the back of the sample is imaged by a camera. **(d)** The corrected focus within the sample allows for optimal photostimulation with the neuronal response recorded by the glass electrode. A representative image of the focus as viewed from the camera: **(e)** A broadened focus; **(f)** Restored focus after encoding a corrective wavefront on the laser.

After calibrating the system with optical materials of known optical aberration (see Supplementary Material 1), we proceed to optimize the laser focus through fixed brain tissues. Figure 1 shows a schematic of this experiment starting with a graphical illustration of a cortical slice adapted from Ramón y Cajal (1909) (Figure 1a). We fixed 100 and 300 μm thick parasagittal brain slices from 15 to 19 day old Wistar rats (see Supplementary Material 2 for brain slice preparation). A thickness of 100 μm was chosen since we normally patch cortical neurons between 50 and 100 μm deep within a 300 μm thick brain slice for electrophysiology experiments. On the other hand, we also fixed 300 μm thick slices to see if we can push our system to propagate our excitation laser through the entire thickness of the brain slice. The slices were placed in between two type-0 coverslips and observed under a custom-built microscope described in Supplementary Material 3. The fixed brain slices were used for prior determination of the Zernike mode correction schematically described in Figure 1b, which illustrates an uncorrected beam propagating through the tissue and Figure 1c showing a wavefront corrected beam via a spatial light modulator (SLM).

**Figure 2:**
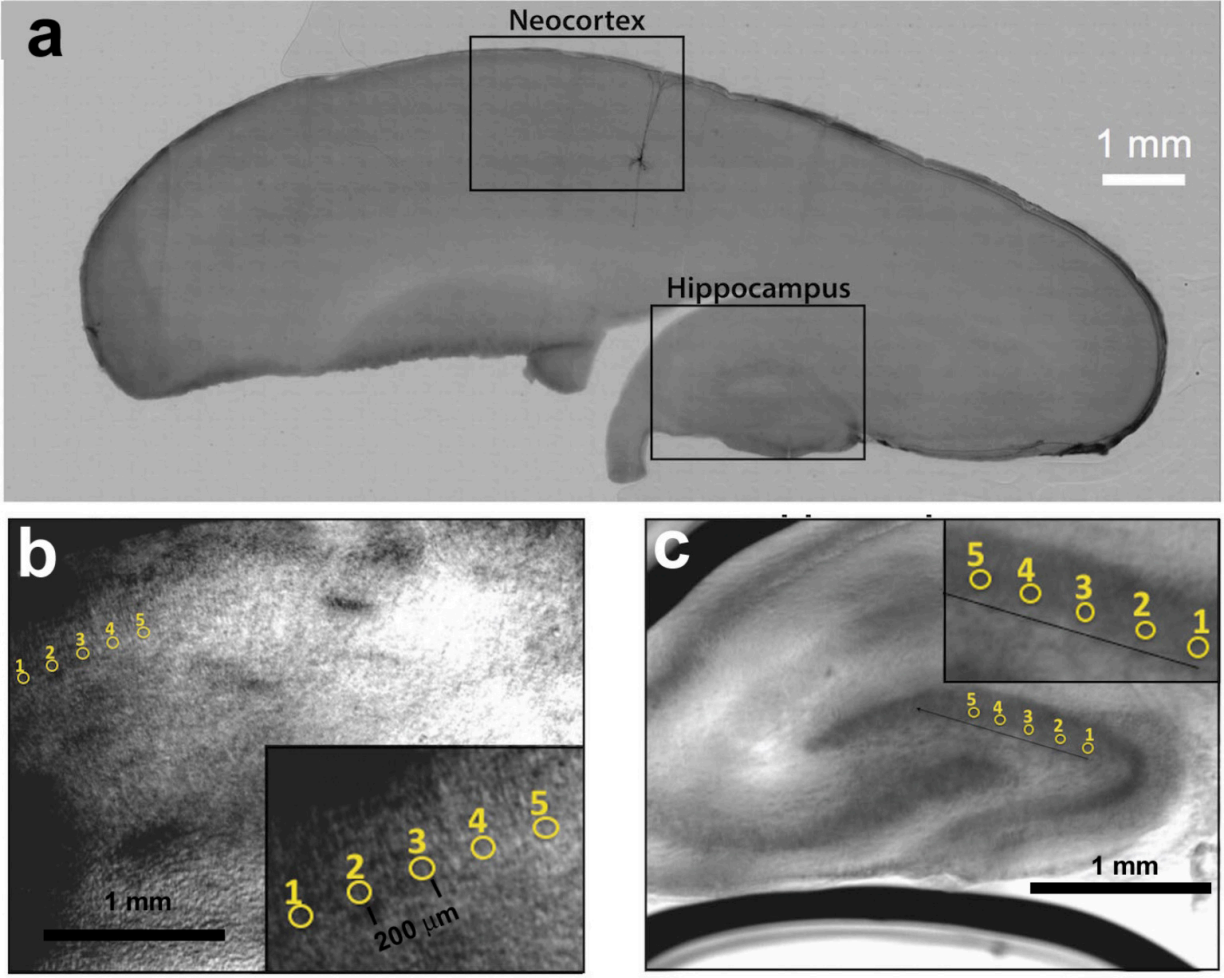
**(a)** Parasagittal rat brain slice indicating the neocortex and hippocampus. A single biocytin-filled Layer 5 pyramidal neuron shows the typical orientation of similar type of neurons in the neocortex. **(b)** Five positions within the neocortex and **(c)** five positions within the hippocampus from where optimization data was obtained.

To derive the corrective wavefront, an iterative algorithm was applied to maximize the beam intensity through the slice of the brain tissue. We find corrective wavefronts on two key cortical regions in the brain slice, namely the neocortex and the hippocampus. These regions are frequently used for *in vitro* studies of neuronal function. Sets of locally optimized phase corrective wavefronts in five (5) separate positions at around 200 μm apart were recorded (see Figure 2). The metric used to retrieve the aberration correction was taken from the quality of the beam focus positioned at the bottom of the tissue. The focus was imaged onto a camera and the quality of the beam was maintained by imposing a digital pinhole on the image of the focus. As the incident laser propagates through the sample, the aberrations reduce the intensity of the focus. Figure 1e shows a representative image of a distorted focus after traveling through a brain tissue. We iterate a finite number of Zernike modes (Noll Zernike terms, NZT = 1–15) over phase multipliers (or coefficients), which changes the wavefront of the incident light and influence the intensity of the laser spot. Our “hill-climbing” algorithm incrementally alters the coefficient of each mode in an iterative loop, and sequentially incorporates only the modes that increase the intensity of the focus. This correction is achieved by encoding the phase pattern onto the SLM, and actively tracking the net intensity within the digital pinhole after each change. The algorithm converges to a phase pattern producing a focal spot with high total intensity and the coefficients of the Zernike modes corresponding to the correction are recorded. This method allows us to select the optimal configuration of Zernike modes without resorting to pixel-based and arbitrary phase manipulation (Ji et al., 2010). Our algorithm took ~2–5 min and ~10–20 min for 100 and 300 μm thick slices, respectively. Figure 1f shows a representative image of the focus after wavefront correction.

### 2.2. Photolysis of caged neurotransmitters

Figure 1d shows the schematic of how we improved the efficiency of uncaging by correcting the wavefront using the Zernike modes derived in the previous section. Our custom-built wavefront shaping 2P microscope (Supplementary Material 3) is built around a DIC microscope (Olympus BX50) to visualize the neurons and implement a whole-cell patch with a glass electrode. Phase contrast imaging degrades at depths lower than 150 μm, while neurons shallower than 50 μm are degraded given that their dendrites are severed during slicing. Hence, we chose to patch onto cortical layer 5 pyramidal neurons between 50 and 100 μm deep within the parasagittal brain slice (300 μm thick).

The electrode contained an internal cellular solution as well as Alexa Fluor dye to visualize the dendritic tree (see Supplementary Material 2 for details of our sample preparation). After patching, the dye was allowed to diffuse into the neuron for 20–30 min before imaging the neuron using our custom-built 2P microscope (Supplementary Material 3). A 3D image of the neuron and its dendrites helps us to identify the sites for photolysis (or uncaging) of caged neurotransmitters. To acquire the 3D image, we set the laser wavelength to 800 nm with 7–15 mW laser power. Image stacks of 800 × 800 pixels in a single plane were generated by imaging individual planes at 1 μm increments along the *z*-axis. We used ImageJ (National Institute of Health) for 3D visualization.

From the 3D image, we identified sites for 2P glutamate uncaging along the dendritic tree and positioned a single uncaging spot via holographic encoding as described in our previous work (Go et al., 2013). MNI-caged glutamate (20 mM; Tocris Bioscience) was locally perfused through a micro-pipette introduced with a constant pressure (1.0 kPa). The laser pulse (2 ms) was controlled via a Uniblitz VS25 shutter (Vincent Associates). Two-photon uncaging was performed at 720 nm wavelength with average powers of 20–25 mW measured at the focus. While we typically locate neurons at 100 μm deep, we positioned uncaging sites to depths between 50 and 150 μm depending on the extension of its dendrites. For imaging, the glutamate-puffing micropipette was kept out of the imaging field of view and no positive pressure was applied to the pipette to avoid premature uncaging.

The glass electrode used to fill the cell with Alexa dye was also used as the recording electrode. Recording of excitatory postsynaptic potentials (EPSP) was done in current clamp mode using a MultiClamp 700B amplifier (Molecular Devices). Only cells with a stable resting membrane potential from the start of the patch were chosen for recording. Analysis was done using *Axograph X* (Axograph Scientific). Peak currents and voltages were calculated by averaging 10 trials and Student's *t*-test was used to determine statistical significance.

## 3. Results

### 3.1. Correcting light though brain slices

Using fixed brain slice samples, we aimed to reduce the complexity for wavefront correction by identifying a few Zernike modes appropriate for optimizing the focus through different regions of the brain tissue. Fixation changes the scattering properties of the brain tissues, which can affect the isotropic scattering properties of extracellular matrix. However, the organization of the neurons and neurites within the cortical tissue is maintained. We observe that in each of the ten (10) different positions in the sample, the hill climbing algorithm successfully produced an optimal focal spot after propagating through the sample. The aberration phase maps are made up of Zernike modes without the need for tedious phase mapping (pixel by pixel) as demonstrated by Čižmár et al. (2010).

**Figure 3:**
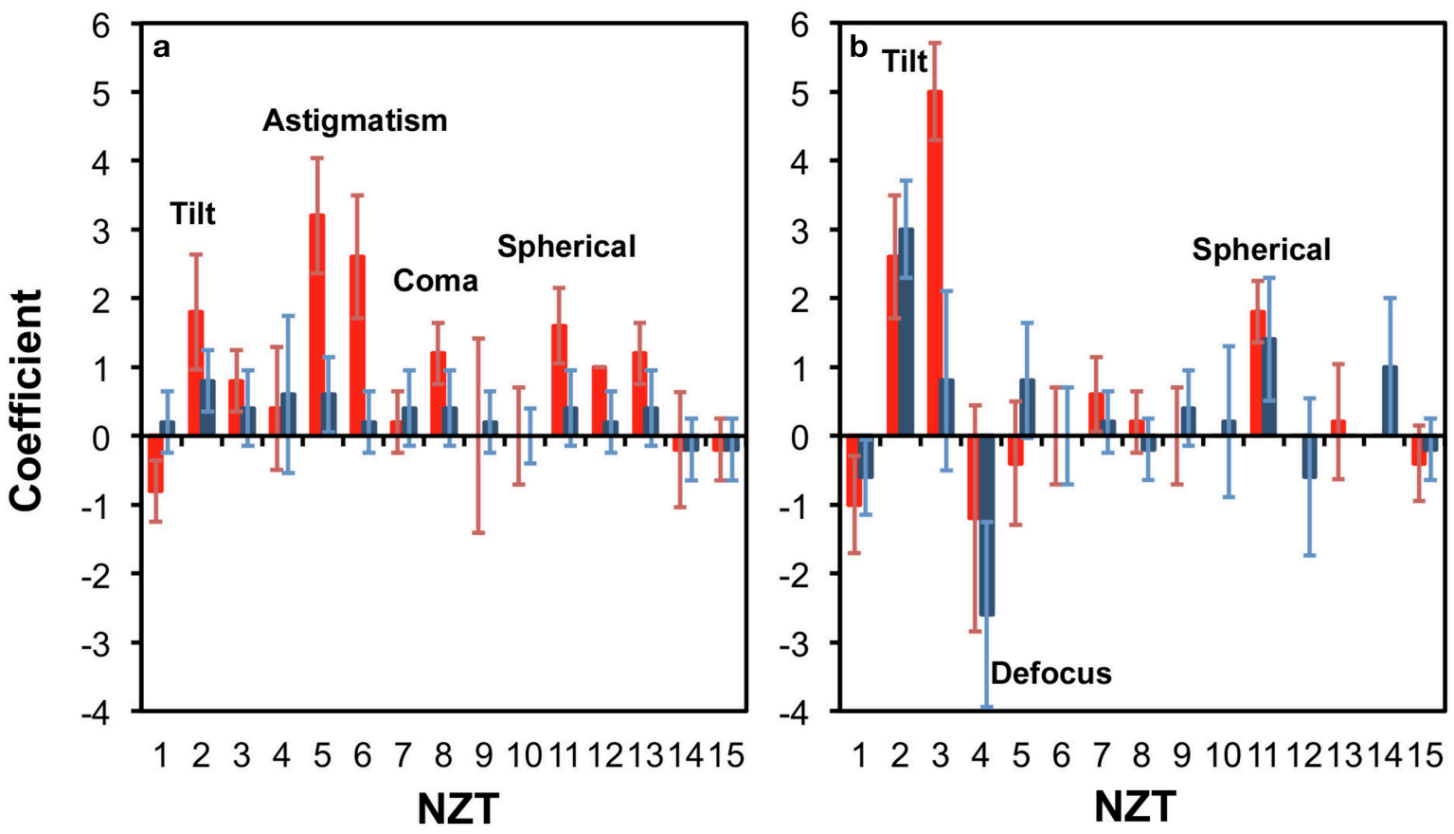
**Zernike modes for correcting light propagation through a (a)** 100 μm; and **(b)** 300 μm thick parasagittal brain slice of rat. The relevant regions were neocortex (red) and hippocampus (blue).

Figure 3 presents the corrections from the five (5) positions in the neocortex (red) and the hippocampus (blue) at two different tissue thicknesses of 100 μm (Figure 3a) and 300 μm (Figure 3b). For each trial (position in the sample), the 30 best corrections corresponding to the 30 highest intensities of the focus are obtained from the hill-climbing method. We select Zernike modes that consistently contribute to the best corrections. Such modes are those that exhibit clear polarity with slight variances (e.g., NZT = 5, coefficient = 3 ± 1). Zernike modes that have large variances spanning to both positive and negative coefficients (e.g., NZT = 10, coefficient = 0 ± 1) are instead interpreted as local correction modes that cannot be generalized for utilization within the studied region.

Figure 3a shows that at 100 μm thick brain slice within the cortical region, astigmatism (NZT = 5 and 6), coma-x (NZT = 8) and spherical aberration (NZT = 11) converge to consistent corrections, and can be identified as a corrective modes (with coefficients ~3, 1, and 2, respectively). This is consistent with Wang et al. (2015) who recently reported the same modes (astigmatism, coma and spherical) dominating their corrective wavefront, obtained via guide star sensing. The tilt terms (NZT = 2 and 3) also converge to a positive coefficient, which is due to the surface flatness of the tissue as well a systematic tilt of the stage. With thicker tissue (300 μm), Figure 3b shows that spherical correction (NZT = 11) converges for both neocortex and the hippocampus, while tilt correction is more pronounced. The other terms do not converge clearly compared to that of the 100 μm thick sample.

### 3.2. Efficient two-photon photolysis with wavefront correction

From the previous experiment, we observed that each local correction relied heavily on just two or three out of the 12 available (non-tilt) Zernike modes. We then proceeded to use this prior information on wavefront correction for optimizing 2P photolysis (uncaging) of caged glutamate at specific dendritic locations of neurons embedded within the brain slice. When released, the uncaged glutamate binds to receptors on the postsynaptic membrane causing a net flow of positive ions into the cell (Callaway and Katz, 1993; Denk, 1994). The resulting voltage response or EPSP can then be measured via the patch-clamp recording from the soma.

The non-linear absorption involved in 2P photolysis is similar to 2P fluorescence excitation where two low-energy (near-infrared) photons are required to release a caged neurotransmitter. A femtosecond (fs ~10^−15^s) pulsed laser focused by a high numerical aperture objective lens is necessary to provide a sufficiently high spatio-temporal photon density to bring about localized 2P photolysis within a small focal volume. When the laser propagates through the brain tissue, aberrations spatially broadens the focus and the efficiency of 2P photolysis is reduced. Thus improving the focus by wavefront correction increases the uncaging efficiency, which is associated to more caged neurotransmitters released as a result of a tighter focus.

**Figure 4:**
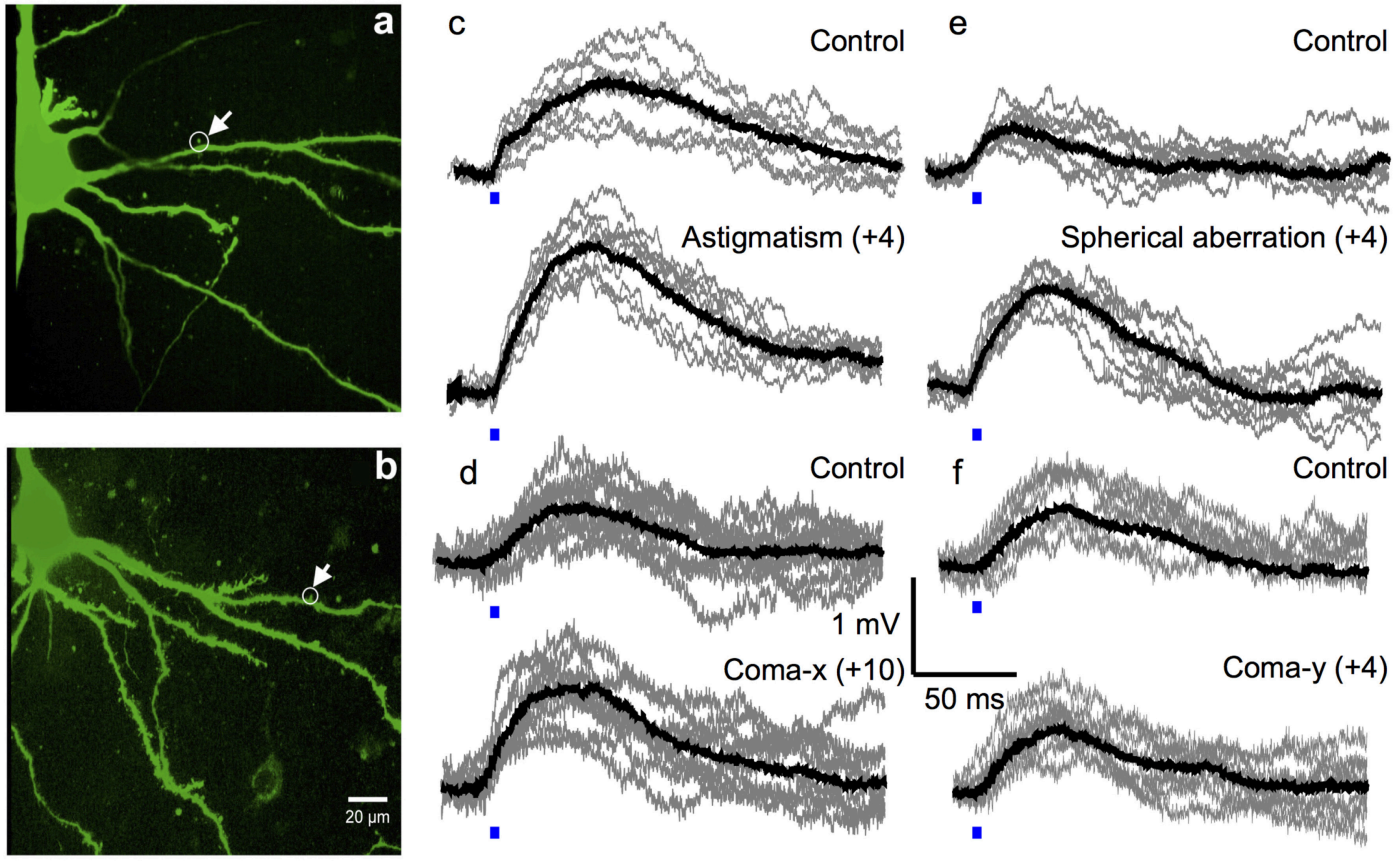
**Optimal 2P photolysis of caged glutamate with wavefront correction. (a,b)** Representative 2P flattened stack images of neurons showing location of the dendritic spine targeted for 2P photolysis. Average light evoked EPSP responses (black traces) each superimposed on ten single episodes (gray traces) measured in a layer 5 pyramidal cell, showing example of wavefront correction before (top panel) and after (bottom panel) for **(c)** astigmatism, **(d)** coma-x, **(e)** spherical aberration, and **(f)** coma-y. Scale bar 20 μm.

**Figure 5:**
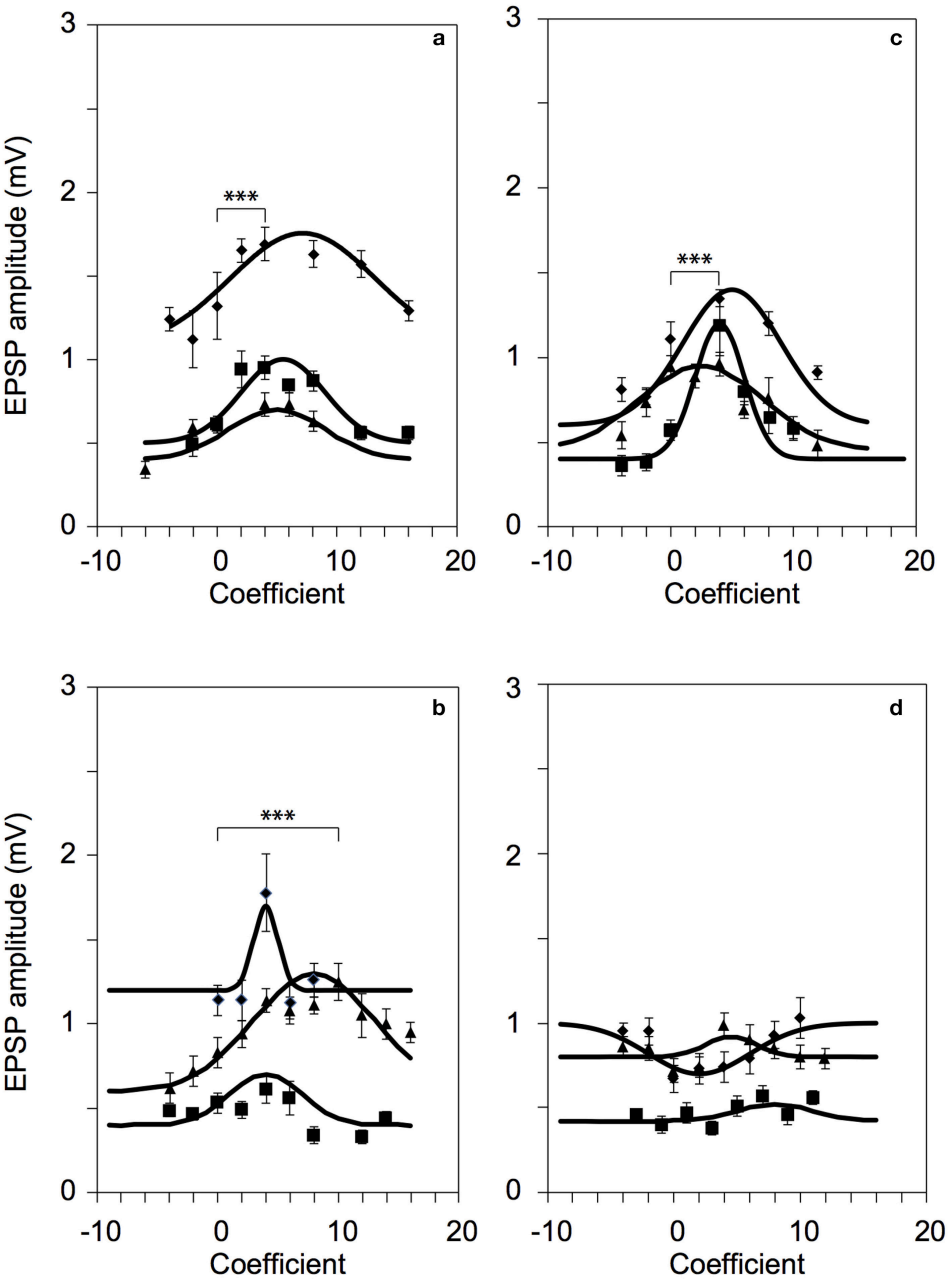
**Successful 2P photolysis optimization using pre-predetermined Zernike modes**. Responses from twelve (12) L5 pyramidal neurons for: **(a)** astigmatism, NZT = 5 (*p*-value = 0.00015, diamonds) **(b)** coma-x, NZT = 8 (*p*-value = 0.00077, triangles), and **(c)** spherical aberration, NZT = 11 (*p*-value = 0.00039, squares). **(d)** As expected, correction with coma-y did not produce optimized responses. ^***^p < 0.001.

To optimize the efficiency of 2P photolysis within the brain tissue, the laser was encoded with the pre-derived Zernike modes in addition to lens and prism functions to position the focal spot in 3D (Go et al., 2013). Figure 4 shows representative images of Layer 5 cortical pyramidal neurons and corresponding EPSP responses following 2P photolysis. Figures 4a,b show positions of uncaging sites along basal dendrites with respect to the soma. The neuron's cell body is located around 50–100 μm from the surface of the 300 μm thick parasagittal brain slice. The neuron's dendritic tree extends in three dimensions and may spread deeper into the tissue. We use the Zernike modes identified for a 100 μm thick tissue, namely astigmatism (NZT = 5) (Figure 4c), coma-x (NZT = 8) (Figure 4d) and spherical aberration (NZT = 11) (Figure 4e). Control traces shown in Figure 4 are traces with no wavefront correction or when the Zernike coefficient = 0. EPSP responses with wavefront correction in Figure 4 are those with the largest EPSP response. Setting the astigmatism coefficient = +4 results in a maximum EPSP of 1.69 ± 0.07 mV as compared to control with 1.32 ± 0.20 mV (*p*-value = 0.00015). Setting the coma-x coefficient = +10 results in a maximum EPSP of 1.25 ± 0.11 mV compared to control 0.83 ± 0.09 mV (*p*-value = 0.00077). Setting the spherical aberration coefficient = +4 results in a maximum EPSP of 1.18 ± 0.17 mV compared to control 0.57 ± 0.06 mV (*p*-value = 0.00039). To show a negative result, we encoded the wavefront for correcting coma-y (NZT = 7) and as expected no consistent optimization (*p*-value = 0.42) was observed for the three trials (Figure 4f). Figure 5 summarizes the changes in EPSP amplitude as a function of Zernike coefficient. As predicted, unimodal optimization was observed for positive coefficient values in every animal trial. Note that the coefficient refers to the strength of correction and may vary depending on the location of stimulation. Each of the twelve (12) traces correspond to a different Layer 5 pyramidal cell from different animals, and each point is the average of 10 measurements of the peak EPSP. The error bars represent the standard deviation.

## 4. Discussion

Traditionally in astronomy, adaptive optics (AO) systems are employed to remove static aberrations from dynamic aberrations in the atmosphere. These optical aberrations are decomposed into Zernike modes, which are part of an orthogonal basis set. In recent years, AO technologies have been gradually adopted by biomedical scientists to remove the optical distortion caused by refractive index heterogeneity in biological tissue. Many of the tissue-based AO optimization strategies borrow heavily from astronomy techniques. Adaptive optics in astronomy typically deals with fast-changing small refractive index fluctuation. However, in tissue imaging, a much larger variation in the refractive heterogeneity with less rapid fluctuation is expected (not accounting for blood flow). Previously, Rueckel et al. (2006) and Zeng et al. (2012) began establishing tissue specific aberration optimization techniques based on fluorescence signals and Zernike modes. And more recently, Park et al. (2015) have demonstrated imaging brains through an intact skull. These techniques show the great potential in this field, however they all rely on complex measurement and correction of the scattered wavefront.

Using Zernike modes, we limit the optimization down to 15 modes, which is far more reduced compared to adjusting the phase of an array of *N* phase-shifting pixels (where *N* ~ 3,228) (Vellekoop and Mosk, 2007). We demonstrate that local wavefront corrections can be made using this finite set of Zernike modes. Furthermore, if we select specific regions within the brain slice, we further reduce the complexity to about 3 to 4 Zernike modes, effectively reducing an *N* dimensional problem to 3 or 4 dimensions. Our experiment essentially shows that light scattering in brain tissues are not totally random but can be deterministic depending on the region of interest.

*A priori* identification of the correction modes is effective for correcting the fundamental aberrations caused by the organization of neurons and neurites on the beam path and prior to the focus. We used fixed brain samples to pre-determine the Zernike modes. While the isotropic scattering properties of the extracellular matrix could be affected by fixing, the organization of the neurons and neurites within the cortical tissue remains intact. Such organizations of cellular structures manifest as modulations in refractive indices that primarily cause rectifiable aberrations on the wavefront of an incident laser. While a full adaptive optics implementation could possibly yield optimal results, our aim is to simplify the correction using a finite set of Zernike modes so we can effectively use it in a time-critical experiment with living cells. During an *in vitro* experiment with fresh brain slices, scaling the coefficients will still be required using only the Zernike modes that we have identified to be significant for specific brain regions. Our 2P uncaging results with wavefront correction using *in vitro* brain slices (Figures 4, 5) were consistent with the results from the fixed slices (Figure 3a), with slight differences in Zernike coefficients. The variations in Zernike coefficients relates to the contrast in refractive index modulations between the cellular structures and the extracellular matrix that distorted the laser before the focus. Aside from the differences in axial position of the focus (e.g., 100 ± 50 μm), we hypothesize that such contrast could also change with the age of the animal. Unfortunately, we don't have enough data to support such claim yet. Nonetheless, we have identified three (3) Zernike modes that improves our uncaging efficiency. The negative result with coma-y aberration shown in Figure 4 was our control experiment as predicted by the results from the fixed slices. In our *in vitro* experiments, we normally orient the brain slice at a particular direction so the apical dendrites of cortical pyramidal neurons are directed along the y-direction. Hence, correcting for coma-y aberration has negligible effect.

While the Zernike modes we used could be specific to the brain regions and depths we studied, the procedure can be extended to different areas of the brain and with other slice preparation (e.g., coronal slices as opposed to parasagittal). Moreover, a similar procedure can also be used to map out aberrations for *in vivo* observation of the brain. Hence, pre-derived corrective Zernike modes for different types of time-critical experiments can be obtained to allow for direct optimization of the incoming wavefront, as an alternative to “on-the-fly” wavefront sensing and adaptive wavefront correction.

## 5. Conclusion

The brain consists of a diverse set of neurons and its neurites that are organized to form networks. These cell structures differ in refractive indices as compared to the extracellular matrix at the background. When viewing a brain slice under a phase contrast microscope, such differences in refractive indices visually aid in navigating through various brain regions. Hence, the propagation of a focussed laser beam through a brain slice can be treated as deterministic and can be associated with a transmission matrix, which manifests as an optical aberration that affects the quality of the focus of an incident coherent light. Since different brain areas have unique organizations of cell structures, we can therefore correlate optical aberrations with specific regions in the brain. We have tested our hypothesis by pre-determining a set of Zernike modes to correct for optical aberrations in specific regions in the cortex. We have shown that our pre-determined set of Zernike modes encoded on the focussed laser beam can significantly improve the focus as measured by a patched neuron's response following 2P photolysis. Having pre-determined the Zernike modes is advantageous in time-critical experiments where an adaptive search for a corrective wavefront can not be performed.

## Ethics statement

All animal housing, breeding and surgical procedures were approved by the Animal Experimentation Ethics Committee of the Australian National University and conform to the guidelines of the National Heath and Medical Council of Australia.

## Author contributions

JC and VD performed the uncaging experiments. SS, WL, HB, and VD designed the calibration experiments. SS performed the determination of Zernike modes using fixed slices. CS provided the brain tissues and advice on the electrophysiology experiments. SS, JC, WL, and VD wrote the manuscript with inputs from CS and HB.

## Funding

This work is supported by the Australian Research Council Discovery Project (contract no. DP140101555) and National Health and Medical Research Council Project Grant (contract no. PG1105944).

### Conflict of interest statement

The authors declare that the research was conducted in the absence of any commercial or financial relationships that could be construed as a potential conflict of interest. The reviewer MT and handling Editor declared their shared affiliation, and the handling Editor states that the process nevertheless met the standards of a fair and objective review.
