# Supplementary material for: Improving Focal Photostimulation of Cortical Neurons with Pre-derived Wavefront Correction
Source: Front Cell Neurosci. 2017 May 1;11:105. doi: 10.3389/fncel.2017.00105 (PMC5410561; doi:10.3389/fncel.2017.00105)
Supplement: Supplementary file 1 [file Presentation1.PDF]

Supplementary Material: Improving focal photostimulation of cortical neurons with pre-derived wavefront correction

Julian M.C. Choy, Sharmila S. Sané, Woei Ming Lee, Christian Stricker, Hans A. Bachor and Vincent R. Daria*

*Correspondence:
Vincent R. Daria:
vincent.daria@anu.edu.au

1 CALIBRATION

To calibrate for systematic aberrations caused by slight misalignments in the construction of the microscope, we first optimized the focus through optical materials exhibiting a known aberration. A stack of microscope coverslips is known to introduce spherical aberration Ji et al. (2010). We increased the aberrations by

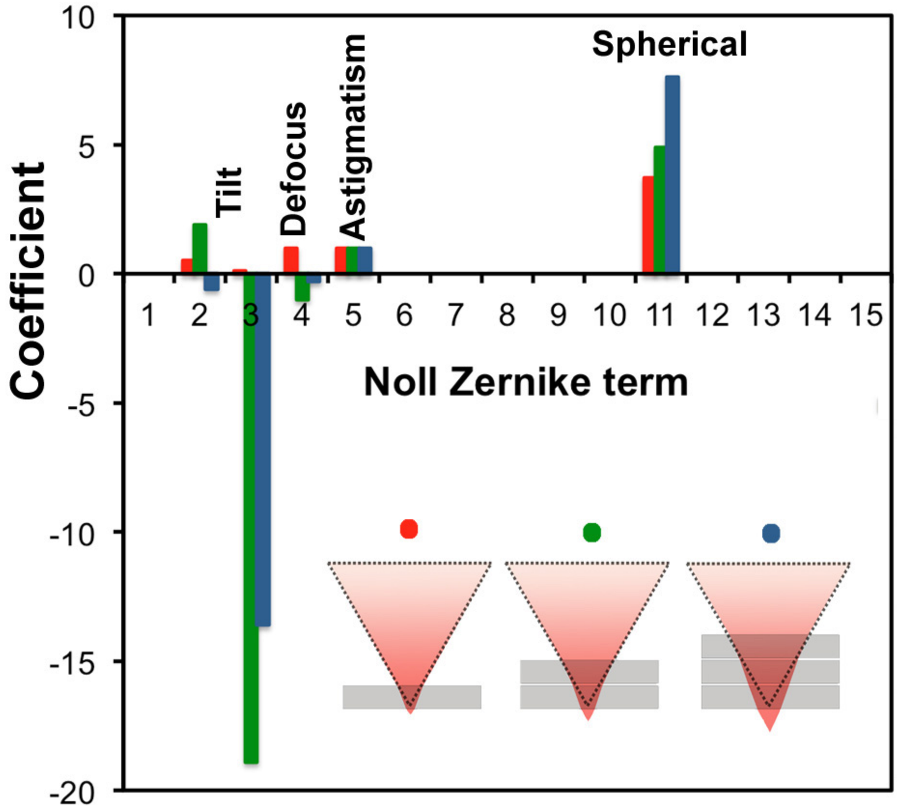

Figure S1. Calibrating wavefront correction via Zernike modes with coverslips as samples. The coverslips introduce known aberrations (e.g. spherical aberration) as depicted in the plot with a positive coefficient (approx. 5-8) for Noll Zernike term NZT=11 which is required to correct for spherical aberration.

stacking microscope coverslips of equal thickness and refractive index without altering the position of the two objectives. This results in the paraxial rays and the marginal rays having a larger path length difference that lead to larger spherical aberrations. In figure S1, we show the imposed wavefront decomposed into its respective Zernike modes for optimizing through 1 to 3 glass coverslips. The coefficients on the y-axis describe the magnitude of wavefront correction using a particular Zernike mode. The Zernike modes that showed a clear improvement in the focus are spherical aberration (NZT=11) and defocus (NZT=4). Figure S1 also shows a systematic astigmatism correction (NZT=5) caused by slight misalignment in the optical system. Note that the coefficient for astigmatism does not change with increasing number of coverslips. Corrections to tilt (NZT=3 and 4) are caused by the angle of the stage with respect to the optical axis. When stacking the coverslips, slight variations in coverslip flatness cause some amount of tilt uncertainty. The rest of the Zernike modes do not contribute to the optimization of the focus.

2 SLICE PREPARATION

We prepared parasagittal brain slices from 15 to 19 day old Wistar rats. We followed the standard procedures for animal experiments in accordance with the methods approved by the Animal Experimentation Ethics Committee of the Australian National University, Australia. The slices were cut with a vibratome (Leica VT1200S) in ice-cold oxygenated artificial cerebrospinal fluid (ACSF) that contained (in mM): 1.25 NaH_2PO_4, 1.0 $MgCl_2$, 125.0 NaCl, 2.5 KCl, 2.0 $CaCl_2$, 25.0 $NaHCO_3$, and 10.0 glucose. Slices were incubated in oxygenated ACSF at 34°C for 30 min and kept at room temperature before being fixed or transferred to the recording chamber. For *a priori* derivation of Zernike modes, we fixed 50 μm to 300 μm thick brain slices (total of 12 parasagittal slices from the center part of the brains from 2 animals). The slices were fixed in 4% paraformaldehyde, embedded in Mowiol 4-88 medium and placed in between two type-0 coverslips. To match with our electrophysiological experimental settings, we chose 2×100 μm thick and 2×300 μm thick slices for *a priori* identification of Zernike modes within the cortical area.

To demonstrate effective photostimulation of neurons, we prepared 300 μm thick brain slices and targeted pyramidal cells in layer V in the somatosensory cortex. The neurons were patched with a glass electrode (resistance: 4-6 MΩ) containing (in mM): 115 K-gluconate, 20 KCl, 10 HEPES, 10 phosphocreatine, 4ATP-Mg, 0.3GTP, 5.4 biocytin, and 0.3 AlexaFluor-488 (Sigma-Aldrich). Recording of the neuron's postsynaptic potentials was performed using a MultiClamp 700B amplifier (Molecular Devices) and analyzed using *Axograph X* (Axograph Scientific).

3 OPTICAL SETUP

Figure S2 shows the multi-functional two-photon microscope setup Go et al. (2013) where a femtosecond pulse Ti:S laser (Coherent Inc. MIRA 900 pumped with 12 W Coherent Verdi G12) is split via a polarizing beam splitter (PBS1). The S-polarized beam is directed to a xy-galvano-scanning mirror setup and used for two-photon imaging. PBS2 couples the S-polarized beam to form a two-photon microscope together with lenses (L4, L5 and L7), dichroic mirrors (DM1) and the photomultiplier tube (PMT, Hamamatsu Photonics, H107201-04) to detect the fluorescence from the sample. On the other hand, the P-polarized laser beam is directed towards an SLM (Hamamatsu Photonics LCOS X10468-02) via a series of mirrors and beam expansion lenses, L1 and L2. A linear combination of Zernike modes is generated by the computer algorithm and displayed on the SLM, which introduces a relative phase delay on the incident light proportional to the brightness of the pixel. The encoded phase map is relayed via a 4f-lens configuration (L3 and L4) to the back aperture of the objective lens (Obj1, Zeiss W Plan Apochromat 40x, NA=1.0).

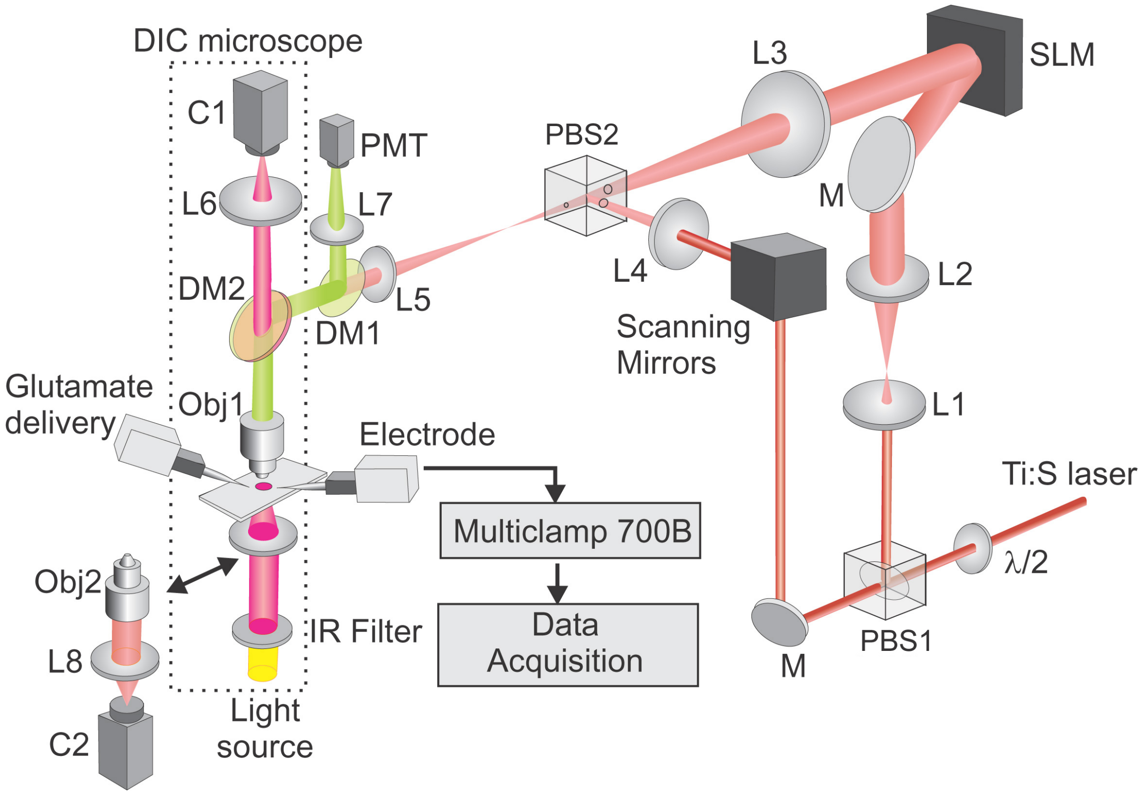

Figure S2. Optical setup showing two Ti:S laser illumination modes: (1) for a two-photon scanning microscope via the galvanometer scanning mirrors; and (2) for a wavefront encoded beam via the spatial light modulator (SLM). Both illumination modes are combined for focal illumination to the sample via the objective lens (Obj1). The microscope is built from a conventional differential interference microscope using camera, C1. For *a priori* derivation of Zernike modes, the focus is imaged by Obj2 and onto camera, C2. Other components: mirror (M); lens (L); half-wave plate ($\lambda/2$); dichroic mirror (DM); photomultiplier tube (PMT); and polarizing beam splitter (PBS).

The appropriate wavefront correction at the back aperture of the objective lens projects a corrected focus at the Fourier plane. The P-polarized beam is also coupled to the microscope using PBS2. For *a priori* identification of Zernike modes, the laser spot focused at the bottom of the brain slice is imaged onto a CCD camera and digitally recorded after passing through another objective lens (Obj2, Olympus 20x), L8 and onto a camera, C2. During electrophysiological experiments with living neurons, the setup at the bottom is replaced with polarized illumination for differential interference microscopy (DIC, Olympus BX50WI) together with L6 and C1. Using a DIC microscope allows for easier patching of the glass electrode onto the neuron's membrane.

REFERENCES

Ji N, Milkie DE, Betzig E. Adaptive optics via pupil segmentation for high-resolution imaging in biological tissues. *Nature methods* **7** (2010) 141–147.

Go MA, To MS, Stricker C, Redman S, Bachor HA, Stuart GJ, et al. Four-dimensional multi-site photolysis of caged neurotransmitters. *Frontiers in cellular neuroscience* **7** (2013).
